# Supplementary material for: Sleep Disturbances and Dementia in the UK South Asian Community: A Qualitative Study to Inform Future Adaptation of the DREAMS-START Intervention
Source: Geriatrics (Basel). 2025 Sep 8;10(5):121. doi: 10.3390/geriatrics10050121 (PMC12452340; doi:10.3390/geriatrics10050121)
Supplement: Supplementary file 1 [file geriatrics-10-00121-s001.zip › geriatrics-3736772-supplementary.pdf]

Supplementary File S1 : Qualitative interview topic guide

UCL

DIVISION OF PSYCHIATRY

DREAMS Widening Access Topic Guide

Interview topic guide: Family Member of Relatives with Dementia from South Asian backgrounds

INTERVENTION CHARACTERISTICS

CONTENT

1. What did you like about the sessions/manual content?
2. What didn't you like about the sessions/manual content?
3. Was there anything in the manual you feel would need to be explained better?
4. Is there any information you felt was missing from the manual? Can you tell me a bit more about that?
5. What did you think about the number of sessions? Were they too many/too little?
6. Is there anything you feel we haven't considered for people with dementia and family carers from South Asian backgrounds? Can you tell me a bit more about that (for example religious beliefs, cultural heritage, family beliefs, language, migration, and access to services).
7. Was there anything you felt wouldn't be useful people with dementia and family carers from South Asian backgrounds?
8. What did you think about the examples and scenarios in the manuals – were they relevant to the South Asian community?
9. There are certain parts of the manual that refer to a 'carer' – do you think of yourself as/prefer being called a carer?
10. Did you/Would you share anything from the manuals with another family member/s? How did you feel?

DELIVERY, CHARACTERISTICS, COMMUNITY SETTING

11. In some cases, we would be delivering DREAMS START with an interpreter. How do you think a family carer who didn't speak English feel about that?
12. We are planning to translate the manuals into Hindi – What do you think about this? Would this be helpful?
13. Do you feel that the culture of the person delivering the intervention is important?
14. Can you think of what might make it difficult for a family carer to attend DREAMS START sessions (e.g., travel, difficulty leaving relative to attend sessions, losing interest or other family commitments)?
15. Can you think of why a family carer might stop coming to sessions?
16. What would people from the community think about you receiving DREAMS START?
17. With any of these issues can you think of how we can adapt and deliver DREAMS START to help?
18. Do you know of any other services like DREAMS START, that are aimed at sleep problems in dementia?
19. Where would you usually come by information about services that are available to you?
